# Reducing Methamphetamine Use in Aboriginal and Torres Strait Islander Communities With the “We Can Do This” Web App: Qualitative Evaluation of Acceptability and Feasibility

**DOI:** 10.2196/58369

**Published:** 2025-07-15

**Authors:** Leda Sivak, Rachel Reilly, Shani Crumpen, Carla Treloar, Rebecca McKetin, Julia Butt, Yvette Roe, Nadine Ezard, Brendan Quinn, Jack Nagle, Wade Longbottom, Clifford Warrior, James Ward

**Affiliations:** 1 Warliparingga Aboriginal Health Equity, South Australian Health and Medical Research Institute, North Terrace, PO Box 11060, Adelaide, 5001, Australia, 61 881284216; 2 School of Psychology, University of Adelaide, Adelaide, Australia; 3 Kaiela Institute, Shepparton, Australia; 4 Poche Centre for Indigenous Health, Department of Rural Health, University of Melbourne, Shepparton, Australia; 5 Centre for Social Research in Health, University of New South Wales, Sydney, Australia; 6 National Drug and Alcohol Research Centre, University of New South Wales, Sydney, Australia; 7 School of Arts and Humanities, Edith Cowan University, Perth, Australia; 8 Molly Wardaguga Research Centre, Charles Darwin University, Brisbane, Australia; 9 National Centre for Clinical Research on Emerging Drugs, National Drug and Alcohol Research Centre, University of New South Wales, Sydney, Australia; 10 Burnet Institute, Melbourne, Australia; 11 Real Drug Talk, Melbourne, Australia; 12 South Coast Aboriginal Medical Service Corporation, Nowra, Australia; 13 Port Lincoln Aboriginal Health Service, Port Lincoln, Australia; 14 Poche Centre for Indigenous Health, University of Queensland, Brisbane, Australia

**Keywords:** Aboriginal and Torres Strait Islander, framework analysis, methamphetamine, process evaluation, qualitative, web-app, app, application, acceptability, feasibility, Australia, primary care, rehabilitation

## Abstract

**Background:**

Preventing and treating methamphetamine-related harm in Aboriginal and Torres Strait Islander populations is a significant challenge for health care services. Digital health care may offer opportunities to support individuals and families in ways that complement existing methamphetamine treatment options. This study responds to a community-identified priority as Aboriginal Community Controlled Health Services identified methamphetamine use as a key concern and sought support to respond to the needs of people who use methamphetamine and their families.

**Objective:**

This paper reports on a process evaluation of the web application’s acceptability and feasibility when used by clients and clinicians in residential rehabilitation services and primary care. This study is part of a larger project entitled “Novel Interventions to address Methamphetamine use in Aboriginal and Torres Strait Islander Communities” (NIMAC), which seeks to develop culturally appropriate and strengths-based prevention and treatment interventions to reduce methamphetamine related harm. *“*We Can Do This” was a web application developed for Aboriginal and Torres Strait Islander people who are seeking to reduce or stop methamphetamine use.

**Methods:**

Clinicians and clients who had used the web application were recruited through Aboriginal Community Controlled Health Services and Aboriginal residential rehabilitation services in urban and regional Victoria and South Australia. Unidentified usage data was collected from all participants. After using the web application, those who indicated a willingness to be interviewed were contacted and interviewed by phone or in person and asked about the feasibility and acceptability of the web application. The framework method of analysis was used to structure and summarise the resulting qualitative data.

**Results:**

Interviews with 24 clients and 11 clinicians explored the acceptability and feasibility of the web application. Acceptability incorporated the following domains: affective attitude, burden, ethicality, cultural appropriateness, coherence, opportunity cost, perceived effectiveness, and self-efficacy. The evaluation of feasibility assessed barriers and facilitators to the implementation of the program, with a focus on demand, practicality, fidelity, and integration. Results indicated that both clients and clinicians found the web application content coherent, relatable, empowering, and culturally safe. Barriers to using the web application for clients included a lack of internet connectivity and personal issues such as scheduling.

**Conclusions:**

Process evaluation is often under-valued. However, as “We Can Do This” was new, innovative and targeted a hard-to-reach population, understanding its feasibility and acceptability as a clinical tool was essential to understanding its potential. “We Can Do This” is unique as the only evidence-based, culturally appropriate internet-based therapeutic program specifically designed for Aboriginal and Torres Strait Islander people who use methamphetamine. Findings suggest it was both acceptable and feasible as a low-cost adjunct to usual care in residential rehabilitation and primary care settings.

## Introduction

### Background

Preventing and treating methamphetamine-related harm in Aboriginal and Torres Strait Islander populations is a significant challenge for health care services. Regular methamphetamine use contributes to adverse mental health outcomes, including substance use disorder, depression, anxiety, and psychosis [1]. In addition to increased hospital admissions [2], the use of methamphetamine is associated with more complex health and behavioral challenges for young people in corrective custody [3] and damages family and social relationships more broadly [4]. Available data indicates that Aboriginal and Torres Strait Islander people are approximately 2.2 times more likely to use methamphetamine than non-Indigenous Australians [5]. This study responds to a community-identified priority as Aboriginal Community Controlled Health Services (ACCHSs) identified methamphetamine use as a key concern [6] and sought support to respond to the needs of people who use methamphetamine and their families.

### Treatment for Methamphetamine Use Disorder

Both residential treatment facilities and outpatient counseling therapies are used for the treatment of methamphetamine use disorder in Australia [7,8]. Psychological interventions such as cognitive behavior therapy (CBT) and motivational interviewing (MI) have shown modest effectiveness [9,10], while strengths-based, collaborative, culturally safe, and family-inclusive approaches to treatment are recommended for Aboriginal and Torres Strait Islander clients [11]. ACCHSs, which provide comprehensive primary health care, are well-placed to provide services to people who use methamphetamines and their families, but demonstrably effective treatment programs for Aboriginal and Torres Strait Islander people are rare. Digital health care may offer opportunities to support individuals and families in ways that complement existing treatment options.

### Web-Based Therapeutic Interventions

Web-based therapeutic interventions (WBTI) have the potential to extend the reach of conventional treatment for methamphetamine [12]. Our previous work has shown that culturally appropriate, evidence-based WBTI have the potential to improve outcomes on a range of health issues and reduce inequalities in access to health care services [12]. The popularity of WBTI stems in part from their social and geographic reach. Chapman et al [13] have further noted that advantages of smartphone apps and web-based interventions for accessing health-related information include anonymity, increased accessibility, low cost, portability of information, and the ability to provide tailored feedback and support [13]. While digital applications have been developed to support Aboriginal and Torres Strait Islander people to address alcohol and other drug use [14], there currently are no WBTIs designed specifically for Aboriginal and Torres Strait Islander people who use methamphetamine.

### NIMAC Study and We Can Do This

This study is part of a larger project entitled “Novel Interventions to address Methamphetamine use in Aboriginal and Torres Strait Islander Communities” (NIMAC). The first 2 phases of the project focused on gathering quantitative and qualitative data on patterns of methamphetamine use and risk and protective factors for use [15], while the latter 2 phases focused on using that information to inform the development of culturally appropriate and strengths-based prevention and treatment interventions to reduce methamphetamine related harm [16].

*“*We Can Do This” is a web application developed for Aboriginal and Torres Strait Islander people who are seeking to reduce or stop methamphetamine use [17]. It comprises 7 modules incorporating evidence-based therapeutic approaches, reflecting elements of CBT, MI, acceptance and commitment therapy (ACT), and narrative approaches, presented in a culturally relevant format. The web application was developed in partnership with 10 ACCHSs nationally, as has been described elsewhere [17].

“We Can Do This” was the subject of a randomized waitlist control trial in which individuals who self-identified as using methamphetamine could access the web application as a stand-alone intervention in the community [17]. The results of the trial are being reported separately. This acceptability and feasibility study arose from a strong interest from clinicians working with Aboriginal and Torres Strait Islander people who use methamphetamine and their desire to have access to the web application as an adjunct to client consultations. Process evaluations are an essential part of testing complex interventions [18] as they identify why an intervention might work and how it can be improved [19]. Successful implementation requires that an intervention be acceptable to both those delivering and those receiving the intervention. This study sought to understand the acceptability and feasibility of the web application when used in the context of primary health care and residential rehabilitation services, as opposed to when used as a self-directed intervention in the community.

## Methods

### Overview

This study is framed within an Indigenous research paradigm [20]. The project was led by an Aboriginal academic and governed by an Aboriginal research governance group to ensure Aboriginal and Torres Strait Islander oversight, guidance and input into the study design, process and outputs. In line with ethical practice supporting data sovereignty for Aboriginal and Torres Strait Islander communities, all data are owned by the participating ACCHSs. This paper was drafted with reference to the CONSIDER Statement [21], which provides consolidated criteria for reporting of health research involving Indigenous peoples, and COREQ (Consolidated Criteria for Reporting Qualitative Research) [22], which provides consolidated criteria for reporting qualitative research.

### Ethical Considerations

The study was approved by the Aboriginal Health Ethics Committee in South Australia (#04-19-810) and St Vincent’s Hospital Human Research Ethics Committee in Victoria (#241‐19). Informed consent was provided by all interview participants before data collection and included permission for secondary analysis of data. All study data were deidentified before analysis and reported anonymously. While clinician participants were not financially compensated, client participants were reimbursed Aus $40 (US $25) in appreciation of their time.

### Interview Guide Development

The semistructured interview guide incorporated questions addressing each of the domains within the overarching constructs of acceptability and feasibility. Acceptability, a multifaceted construct, incorporates the following domains: affective attitude, how the participant feels about the program; burden, the perceived effort required to use the program; ethicality and cultural appropriateness, the extent to which the intervention is a good fit with the participant’s value system; coherence, the extent to which a participant understands the program and how it works; opportunity cost, the extent to which a participant feels they have to compromise values or benefits to engage in the program; perceived effectiveness, the extent to which the program is perceived to achieve its purpose; and self-efficacy, a participant’s confidence that they can use the program [23].

The evaluation of feasibility assessed barriers and facilitators to the implementation of the program, with a focus on: demand, degree of use and interaction with the program; practicality, the extent to which the program can be delivered within current resource and time restraints; fidelity, whether the program was delivered as designed or whether adaptations were made to fit the service context; and integration, the level of system change required to incorporate the program into the service.

### Sampling, Recruitment, and Data Collection

Building on earlier phases of the NIMAC study [17], perspectives regarding acceptability and feasibility were sought from 2 groups of stakeholders who had been involved in earlier phases of NIMAC: Aboriginal and Torres Strait Islander clients of participating health services, aged 16 years and older, who had used the internet-based program at least once; and clinicians from the same services, who had used the program with clients. Clinicians could include nurses, doctors, counselors, Aboriginal health workers or practitioners, psychologists, drug and alcohol workers, youth workers or social and emotional well-being workers.

Clinicians and clients were recruited by the study team through Aboriginal Community Controlled Health Services and Aboriginal residential rehabilitation services in urban and regional Victoria and South Australia that had participated in earlier phases of the NIMAC study. These existing relationships supported the staged approach of the study. After using the web application, those who indicated a willingness to be interviewed were contacted and offered an interview by telephone, in-person or in writing. All interviews were conducted by telephone and took between 40 and 75 minutes.

### Analysis

Interviews were audio recorded, transcribed, and deidentified prior to analysis. Participants were offered the opportunity to review the deidentified transcript of their interview and provide feedback. Two participants requested transcripts, but none provided feedback. Deidentified transcripts were uploaded into NVivo 12 software (Lumivero) for thematic analysis. The interview guide provided a framework for deductive coding, as acceptability and feasibility factors were organized under these categories. Themes that emerged inductively outside of this framework were coded separately. The framework method of analysis was used to structure and summarize the data [24]. Framework analysis comprises five steps: (1) familiarization with the data, (2) identification of a thematic framework, (3) indexing of study data against the framework, (4) charting the indexed data, and (5) mapping and interpretation [25].

Analysis was carried out by 3 researchers, with all data being coded thematically by at least 2 researchers in an iterative process of coding, discussion, and cross-checking. As 2 of these researchers were non-Indigenous, community-based researchers and Aboriginal and Torres Strait Islander members of the investigator team also reviewed the analysis to ensure that findings were expressed in culturally appropriate ways.

## Results

### Participants

Participants in this study included 24 Aboriginal or Torres Strait Islander participants and 11 clinicians who had used the web application. A total of 14 Aboriginal or Torres Strait Islander participants female and 10 were male and ranged in age from 17 to 58 years. Clinicians included a youth worker, 2 psychologists, 3 peer workers, and 5 alcohol and other drug counselors. The following codes are used to identify: P=person with experience, also referred to as “clients”; C=clinician; and W=peer worker.

### Acceptability

#### Affective Attitude

Affective attitude refers to how a participant feels about the program being evaluated. There were several examples of positive affect. As one clinician stated:

*I had a good feeling that there was this kind of app out there available for clients. So going through it, I was like “Yeah, this is looking pretty good.”*

[C2]

Not all participants had clear expectations of the web application, and expressed wariness:

*I was a bit wary of it at first, to be honest, and when I went through it, I thought, “You know what, this is pretty good” and yeah, I actually thought it was better than what I thought it was going to be.*

[C4]

Another participant stated:

*At first I was like, “This is not going to work.” And then after a while, after a couple of goes at it, I was like “Oh yeah, this is actually helping.” And then I got used to it and I enjoyed it.*

[P38]

*At first I was like, “This is not going to work.” And then after a while, after a couple of goes at it, I was like “Oh yeah, this is actually helping.” And then I got used to it and I enjoyed it.* [P38]

Another said, “I wasn’t sure what to expect at first, but yeah, it was really good” (P153). Some participants appeared satisfied:

*I was hoping that the app would help me on the way and it definitely support me in that direction. I went into the app knowing that I needed to try a lot of things to cut back and get off it [methamphetamine].*

[W3]

Some participants felt challenged by the content of the web application. As one client commented, “Sometimes it hit a little too close to home, what these people are saying” (P184). Others, who initially felt scared about the web application, found the video stories relatable and true to life, “It’s good with the videos and stuff – they do hit home with me. I could relate” (P190).

Qualities of the web application associated with positive affect included that it focuses on Aboriginal and Torres Strait Islander people, is easy to understand and straightforward to use, is available at home, and is nonintrusive. Overwhelmingly, participants found the graphics, including the themes and colours, inviting and relatable. One clinician reported, “I thought there was a good Aboriginal theme going through, which is important” (C4). In relation to the films, another participant suggested:

*It’s good to have other people’s comments and their addiction included in it, so you can have a little look and see what we relate to and what we don’t, and how it confronts us*.

[P158]

#### Burden

Participants were equally divided between those who experienced time pressures as a barrier to engaging with the web application, and those who either found it easy to prioritise using it or had experienced no time barriers. More significantly, several participants reported that a lack of internet connection presented a barrier to using the web application at times, and 2 reported having trouble signing in.

A facilitator of use identified by 2 participants was the support of nonjudgemental peer-workers or clinicians who encouraged them to use the web application or helped them to log in. Several participants described features of the web application that they found helpful, such as the flexibility of being able to use it privately, anywhere, any time; being able to access the content in any order; and receiving notifications and reminders from the web application itself.

For similar reasons, most participants reported that it was not an effort to use the web application, although 1 participant did report that it was “emotionally challenging sometimes” (P190). Another suggested that “If we are putting ourselves in difficult situations to use the meth, it shouldn’t be difficult to use the web app” (P200). Although 2 participants found the web application “too wordy” (P142, W1), the accessibility of the language used in the web application was highlighted as a strength of the web application by most participants who responded to this question (n=12).

#### Coherence

All 12 participants who commented on coherence of the web application agreed that the content made sense and was clear in its purpose, with 1 client commenting, “The content of information provided was helpful and clear in how it was meant to help people” (P165). Another participant commented:

*What I took from it, it was self-motivated. So being given and being aware of certain tools, creating that support network and prompting us to think of some supports that we could put down on them that we could call up. It really made sense.*

[W3]

Only 2 clinicians commented on the therapeutic alignment of the web application with their existing practice. Both could see the value of the web application, with 1 clinician noting that the web application will complement a range of therapeutic approaches, given that “There’s no one-fix therapy or approach” (C1). The other clinician, who used a combination of motivational interviewing and harm reduction, commented that the web application “aligned pretty well” (C2).

#### Perceived Effectiveness

A total of 7 participants (including 2 peer workers) commented on the effectiveness of the web application for reducing their methamphetamine use, with almost all finding the web application effective. For example, 1 client observed that the web application “helped when I was craving; it helped at that time when I didn’t have a support person to turn to – I just turned to the app” (P38). Likewise, a peer worker commented:

*The app was really helpful for me, because once I tuned into the app, and I was able to go through and read everything that app had to offer in terms of the modules and the videos and it really helped me. …It really made me think about what stage of change I am at. … It really helped me therapeutically, it helped me.*

[W3]

A total of 5 participants commented on the effectiveness of the web application for avoiding situations where they might feel stigma. In contrast, 1 participant shared that the web application was less helpful than expected, especially as the cravings module increased their experience of cravings.

Furthermore, 5 participants observed that the web application was also helpful for making changes in other areas of their life, with 1 peer worker commenting:

*It really helped me to just really reach out to people when anything was going wrong. It didn’t have to be about ice or anything. I’ve just known that I’ve got some people in my corner and how much they really mean to me.*

[W3]

A client, who noted that the web application helped with their breathing, anxiety and fitness commented that, “It’s mentally empowering, if that’s the word. It helps your mental side” (P54).

#### Ethicality

Most participants were content with the ethicality of the web application. As a client explained, “I felt really safe to just be open and honest on there” (P190). However, one clinician was concerned about participant mental health due to the self-led modality of the application. They suggested incorporating a question function to further support the social and emotional well-being of individuals using the application. Other participants raised concerns around the collection and use of personal data and data security. The majority of participants felt comfortable to raise concerns about the application with a clinician or the research team.

#### Cultural Appropriateness

The web application was found to be culturally appropriate with the inclusion of Aboriginal and Torres Strait Islander people, language, artwork, images, and fonts as relatable for many participants. One client stated:

*It didn’t seem institutionalised. It did not have that government smell about it… You could tell that it is real people trying to help other people, and it’s – it’s real.*

[P190]

Whilst 4 out of 10 participants were unsure of the extent to which the web application aligned with their value system, others felt it was a good fit. For example, one peer worker said:

*It really did fit with my values. And not just my values, my community’s values as well, about mob trying to break this cycle. It’s such an important app. And it really made me feel like I was part of something. It sat with my values really well.*

[W3]

#### Opportunity Costs

A total of 10 participants commented on the opportunity costs associated with the web application, with all observing that there was no need to compromise their other commitments to engage in the program. The flexibility of the web application was especially helpful in this regard, as one client commented:

*It was good in a sense that you could just do it whenever and, if you did have another commitment, it didn’t have to interfere with that because you could just do it afterwards or beforehand.* [P158]

#### Self-Efficacy

In total, 9 participants commented on their confidence using the web application, with almost all feeling immediately confident. In contrast, 1 participant needed to engage with the web application more than once to build confidence with it:

*At the start, I felt sort of like a little bit just in unfamiliar territory. Once I had the option to go back into stuff, it made me a lot more confident to go back in and just give the questions a second crack and read them again and really make sense of it. Just going over them the first time it didn’t really sink in. But when I had an option to go back in and check them out, and go back into ones that sort of sparked my interest more, that really made a lot more an impact, sunk in more*.

[W3]

Furthermore, 8 participants observed that the web application did not require any new skills, with 1 client commenting:

*I think for most people that are familiar with using either phones or any sort of device, it was fairly straightforward how to access the different parts of it*.

[P153]

### Feasibility

#### Demand

Participant use and interaction with the web application varied. Duration and frequency with which an individual recounted using the web application were between 10 and 60 minutes each time, 1‐5 days per week or 2‐4 times in total. Furthermore, 6 out of 10 clients and 1 clinician reported using the application outside of counseling sessions. However, clinicians also described using the web application with clients before giving “them the opportunity to do it themselves” (W1). Participants accessed the web application by desktop computer, tablet, or mobile phone.

#### Fidelity

Fidelity refers to whether participants found they used the web application as designed and suggested by the researchers, or whether they made adjustment to it to suit their own personal or organizational (in the case of clinicians) context. Very few participants responded to this question. The 2 who did respond suggested that they “self-explored” (P165) the web application, or “did it in my own time” (P200), reflecting the flexibility that was intentionally built into the web application design. Given this flexibility, the lack of responses to this question could indicate that the question of fidelity was less relevant to participants’ experiences of using the web application.

#### Integration

Similarly, only 2 clinicians commented on integration, or the systems changes required to incorporate the web application into the service. One commented that due to time constraints within existing service delivery, using the web application with clients had been undertaken opportunistically thus far and that dedicating time for using the web application with clients would make their approach more strategic.

#### Practicality

Most participants reported that they had the equipment they needed to use the web application. The remainder either did not respond directly to this question (n=5) or reported that a lack of reliable internet was a problem for them (n=2). One participant described having intermittent internet but not finding this a problem as receiving text messages from the web application meant that they “could still be reminded of those things” (P153). One clinician reported that using the web application on a tablet was preferable to using it on a desktop computer as it facilitated a more relaxed, conversational setting and they saw their role as supportive rather than directive:

*We’ll share it together and then I can give it to them and they’re in total control of what’s happening with it. I’ll support them with utilising it but they’re in control whether they use it and what they do with it.*

[C1]

Out of the 21 participants who responded to questions about navigation within the web application, 1 participant had trouble going back one step once they were in the module, and 1 peer-worker suggested there were “too many options in some modules” (W1). Another peer-worker had trouble logging people in initially. However, the majority of respondents to this question had no trouble with navigation, and one clinician stated:

*What I liked the most, the interface and the way it controls was very user friendly, especially to those ones who aren’t necessarily the most tech savvy.*

[C5]

Participants varied in terms of whether they went through each module sequentially or in random order; however, most seemed to go through all the modules at least once, then return to those they found most useful. For example, one participant said:

*I went from the first one to the last one. And then, like I said, the option of being able to go back into them. Then I picked the ones that I really was interested in.*

[W3]

A total of 11 participants gave feedback on the practicality of finding time within their routines to use the web application. Of these, one said they could not find the time. Again, flexibility was highlighted as a facilitator for people finding time to use the web application. A participant stated:

*They just said, ‘You can just sit on it anytime you want. You can use it on the bus.’ Because I told them I use public transport. And they just more or less helped me identify all this time I do have to use it*.

[W3]

Another stated:

*Just that it was something that could be there all the time and I could do it when I had time in between other commitments that I had*.

[P153]

Another suggested that finding time to use the web application was harder at first, “But once I started using it, it only took 10‐15 minutes each time” (C1).

## Discussion

### Principal Findings

The results of this study demonstrate that clinicians and clients generally found the “We Can Do This” web application to be both acceptable and feasible when used in the context of primary health care and residential rehabilitation services. *“*We Can Do This” was generally found by both clients and clinicians to be coherent, relatable, empowering, and culturally safe. In particular, study participants valued the flexibility of the web application and noted how it reduced the stigma they sometimes felt in seeking help in other ways. They also liked the video vignettes with Aboriginal and Torres Strait Islander actors and the ability to share or print the module summaries. Challenges with the web application in its tested form included difficulty signing in and barriers with internet connections.

Methamphetamine use is associated with higher use of acute health services and reduced use of community-based or primary health care [2]. McKetin et al [2] note that this may also reflect a nonacute service system that is ill-prepared to meet the needs of people who use methamphetamine. In general practice, drug use can be a difficult topic to broach with patients and to tackle effectively. For example, stigma has been identified as a barrier to treatment-seeking [26,27], with 1 in 3 Australians who use methamphetamine reporting experiences of discrimination and 87% expressing self-stigma [28]. Digital interventions can provide a useful entry point for clinicians to start conversations, break down stigma, normalize some of the challenges experienced by people who use methamphetamine, and guide people toward making positive change.

In contexts where access to trained alcohol and other drug counselors may be limited, web applications have the potential to increase the capacity of health services to respond to the needs of people seeking help for methamphetamine use. For example, having shown that the eHealth platform, Cracks in the Ice, improved knowledge and decreased stigma about methamphetamine, Kershaw et al [29] demonstrate that digital information and support platforms play an important role in improving knowledge and reducing stigma. As digital health care becomes more widely used [13], ensuring that Aboriginal and Torres Strait Islander people can access internet-based support that is culturally appropriate is essential to achieving equity in health service access [30]. “We Can Do This” may increase the capacity of mainstream services to better meet the needs of Aboriginal and Torres Strait Islander people who use methamphetamine in culturally relevant and safe ways.

### Limitations

Interview participants in our sample self-nominated to take part, likely favouring those with strong views and opinions. While findings may not be representative of the experiences and concerns of all Aboriginal and Torres Strait Islander communities in Australia or Indigenous peoples in other countries, the congruence between both client and clinician participants suggests the broad acceptability and feasibility of this web app in primary care and residential rehabilitation settings.

Process evaluation is an essential and often under-valued aspect of program evaluation. Given that the “We Can Do This” web application is a new and innovative program, targeting a unique and often hard-to-reach population, understanding how it is implemented, its feasibility, acceptability, and overall attractiveness as a clinical tool are essential to understanding its value and potential.

### Conclusion

The “We Can Do This” web application is unique and innovative, being the only culturally appropriate internet-based therapeutic program developed specifically for Aboriginal and Torres Strait Islander people who use methamphetamine. This web application makes a contribution to the growing suite of digital resources developed for Aboriginal and Torres Strait Islander health and well-being. The findings of this study provide tentative support for the usability of the program in supported health service settings. While future research should explore its effectiveness at supporting a reduction in methamphetamine use in this context, this study suggests that “We Can Do This*”* may provide a valuable adjunct to more traditional counseling methods, at low cost, without significant burden on health services, making culturally appropriate care more readily available to those who need it.

## Supplementary material

10.2196/58369 Checklist 1 COREQ (Consolidated Criteria for Reporting Qualitative Research) checklist.

## Abbreviations

ACCHS: Aboriginal Community Controlled Health Service
ACT: acceptance and commitment therapy
CBT: cognitive behavioral therapy
COREQ: Consolidated Criteria for Reporting Qualitative Research
MI: motivational interviewing
NIMAC: Novel Interventions to address Methamphetamine use in Aboriginal and Torres Strait Islander Communities
WBTI: web-based therapeutic interventions
